# Microbial antibiotic resistance genes across an anthropogenic gradient in a Canadian High Arctic watershed

**DOI:** 10.1093/sumbio/qvae021

**Published:** 2024-08-07

**Authors:** Juliette Provencher, Paul B L George, Mary Thaler, Warwick F Vincent, Caroline Duchaine, Alexander I Culley, Catherine Girard

**Affiliations:** Département de biochimie, de microbiologie et de bio-informatique, Université Laval, 1045 Avenue de la Médecine Québec, QC G1V 0A6, Canada; Centre d’études nordiques (CEN), Université Laval, Québec, QC, G1V 0A6, Canada; Institut de biologie intégrative et des systèmes, Université Laval, QC, G1V 0A6, Canada; Takuvik Joint International Laboratory, Université Laval, QC, G1V 0A6, Canada; Département de biochimie, de microbiologie et de bio-informatique, Université Laval, 1045 Avenue de la Médecine Québec, QC G1V 0A6, Canada; Centre d’études nordiques (CEN), Université Laval, Québec, QC, G1V 0A6, Canada; Institut universitaire de cardiologie et de pneumologie de Québec – Université Laval, 2725 ch Ste-Foy, Québec, QC, G1V 4G5, Canada; Département de biochimie, de microbiologie et de bio-informatique, Université Laval, 1045 Avenue de la Médecine Québec, QC G1V 0A6, Canada; Centre d’études nordiques (CEN), Université Laval, Québec, QC, G1V 0A6, Canada; Institut de biologie intégrative et des systèmes, Université Laval, QC, G1V 0A6, Canada; Takuvik Joint International Laboratory, Université Laval, QC, G1V 0A6, Canada; Centre d’études nordiques (CEN), Université Laval, Québec, QC, G1V 0A6, Canada; Institut de biologie intégrative et des systèmes, Université Laval, QC, G1V 0A6, Canada; Takuvik Joint International Laboratory, Université Laval, QC, G1V 0A6, Canada; Département de biologie, Université Laval, Québec, QC, G1V 0A6, Canada; Département de biochimie, de microbiologie et de bio-informatique, Université Laval, 1045 Avenue de la Médecine Québec, QC G1V 0A6, Canada; Institut universitaire de cardiologie et de pneumologie de Québec – Université Laval, 2725 ch Ste-Foy, Québec, QC, G1V 4G5, Canada; Département de biochimie, de microbiologie et de bio-informatique, Université Laval, 1045 Avenue de la Médecine Québec, QC G1V 0A6, Canada; Centre d’études nordiques (CEN), Université Laval, Québec, QC, G1V 0A6, Canada; Institut de biologie intégrative et des systèmes, Université Laval, QC, G1V 0A6, Canada; Takuvik Joint International Laboratory, Université Laval, QC, G1V 0A6, Canada; Centre d’études nordiques (CEN), Université Laval, Québec, QC, G1V 0A6, Canada; Institut de biologie intégrative et des systèmes, Université Laval, QC, G1V 0A6, Canada; Département des sciences fondamentales, Université du Québec à Chicoutimi, 555 Boulevard de l’Université, Chicoutimi, Québec, QC, G7H 2B1, Canada

**Keywords:** antimicrobial resistance (AMR), microbiology, bioaerosols, microbial mats, lakes, Arctic, anthropogenic contamination

## Abstract

Antibiotic resistance is one of the biggest challenges to public health. While the discovery of antibiotics has decreased pathogen-caused mortality, the overuse of these drugs has resulted in the increased transfer and evolution of antibiotic resistance genes (ARGs) in bacteria. ARGs naturally occur in wild bacterial communities, but are also found in increased concentrations in environments contaminated by wastewater effluent. Although such ARGs are relatively well described in temperate environments, little is known about the distribution and dissemination of these genes in the Arctic. We characterized the ARGs in microbial communities from aerosols, lakes and microbial mats around a remote Arctic hamlet using metagenomic approaches. Specific objectives were to (i) compare ARGs across habitats, (ii) to characterize ARG populations along a continuum of anthropogenically influenced environments, and (iii) to identify ARGs of viral origin. We identified ARGs in all habitats throughout the watershed, and found that microbial mats in the most impacted area had the highest diversity of ARGs relative to uncontaminated sites, which may be a remnant signal of wastewater effluent inputs in the area during the 20th century. Although we identified ARGs predominantly in bacterial genomes, our data suggests that mimiviruses may also harbor ARGs.

**Sustainability Statement**

Although antibiotic resistance genes (ARGs) are a growing public health concern, little is known on their presence and distribution in the Arctic, or of the anthropogenic and natural drivers of ARG emergence and selection in these ecosystems. The North is warming more rapidly than any other area on the globe, which will likely transform ARG dispersal patterns across landscapes, through changes in hydrological or airborne connectivity. This study presents the ARG profile of watersheds around a hamlet in the Canadian High Arctic that hosts a gradient of anthropogenic impact. The present work aligns to the UN Sustainable Development Goals 3 (Good health and wellbeing), 6 (Clean water and sanitation), and 15 (Climate action).

## Introduction

Antibiotic overuse by humans has resulted in a significant increase in the prevalence of antibiotic resistance genes (ARGs) globally (Tan et al. 2018). This is of particular concern because infections that have been historically resolved with antibiotics are increasingly becoming resistant to such treatments and driving a rise in morbidity and mortality, with approximately 5 million deaths attributable to antibiotic-resistant organisms in 2019 alone (Murray et al. 2022). Bacterial resistance to antibiotics is spreading rapidly across the globe (McCann et al. 2019), and appears to persist in environmental reservoirs (Jara et al. 2020). Although some bacteria maintain resistance to antibiotics even without ongoing selective pressure (Tam et al. 2015), the main driver of the spread of resistant bacteria is through human activities such as agriculture and wastewater discharge (Singer et al. 2016, Tan et al. 2018). The spread and evolution of ARGs across bacterial populations are therefore contributing to a global health crisis, and understanding the distribution of antimicrobial resistance in the environment and its drivers is critical to better meet this growing public health challenge.

In temperate environments where land-use and population density are at their greatest, anthropogenic influence is pervasive and has extensive impacts on the introduction and persistence of ARGs in the environment, especially in aquatic habitats (Guan et al. 2022). ARGs in aquatic systems may originate from multiple sources. This includes industrial agriculture, where high ARG counts have been observed in agricultural soils and runoff from animal husbandry (Neher et al. 2020, Keenum et al. 2021, Rothrock et al. 2021). However aquaculture (Su et al. 2017) as well as wastewater effluent are also major inputs of ARGs into freshwater systems (Berglund et al. 2015, Czekalski et al. 2014, Rodriguez-Mozaz et al. 2015, Proia et al. 2018). Freshwater systems impacted by anthropogenic activity are therefore an important reservoir for ARGs.

Although ARGs are more abundant in areas of high anthropogenic activity, they can also be detected in the most pristine aquatic environments. For instance, ARGs have been found in remote Antarctic lakes and microbial mats, which can serve as natural reservoirs for resistant bacteria (Tallada et al. 2022). This is because ARGs also occur naturally as mechanisms of competition within microbial communities (Letten et al. 2021). Yet, ARGs may also be found in these remote habitats due to the long-range transport of resistant organisms from areas of greater anthropogenic influence through bioaerosols (Péguilhan et al. 2023), which have been identified as a major dispersal force for resistant organisms in the environment (Bai et al. 2022, Tao et al. 2022). ARGs may also potentially be dispersed to remote areas via wildlife, especially via migratory animal populations living in proximity of human activity (Allen et al. 2010, Miller et al. 2020). With ARGs becoming more pervasive even in areas of low local anthropogenic activity, there is a pressing need to understand the diversity and distribution of antibiotic resistance in these remote, more sparsely populated areas. Indeed ARGs have been detected in remote polar regions where the overall presence of human-associated microorganisms as well as antibiotic-resistant organisms has increased due to the presence of military, research, and fisheries installations (Hernandez and Gonzalez-Acuna 2016). A study in the Arctic in the Northern Bering Sea showed that ARGs in marine sediments had two primary sources: resistance in human-associated microbes via sewage, and ARGs from natural *in situ* communities (Tan et al. 2018). However, it remains difficult to distinguish ARGs associated with human activity from those that are naturally occurring in polar environments, due to the relative low intensity and density of anthropogenic activity (McCann et al. 2019). As the poles are warming significantly faster than the rest of the globe (Rantanen et al. 2022), and with human activity expected to increase in polar regions, better characterization of antibiotic resistance in these habitats is vital.

Here, we investigated the dissemination of ARGs in a polar environment with a known contamination event that involved the likely introduction of ARGs associated with human activities into the watershed. We assessed the presence, diversity and distribution of ARGs in lakes, microbial mats and aerosols on Cornwallis Island (Nunavut, Canada), including in Meretta Lake, a rare example of a High Arctic lake that underwent anthropogenic eutrophication through wastewater discharge (Antoniades et al. 2011). Nearby lakes on Cornwallis Island have limited or no hydrological connections to Meretta Lake and therefore did not receive wastewater inputs, but may have been subjected to airborne dispersal of ARGs during the same period. We hypothesized that Meretta Lake and its wetlands, which received the greatest input of sewage (Stewart et al. 2014), would contain a greater diversity and relative abundance of ARGs than other lakes in the area that did not receive direct wastewater inputs. To address this hypothesis, we collected samples and environmental data across this exposure gradient to assess the distribution of ARGs. We used metagenomic approaches to identify ARGs in aerosols, lakes, and microbial mats to determine the relative abundance and diversity of ARGs by habitat and in relation to anthropogenic influence. We also explored genomes harbouring ARGs to determine if viruses are a vector of transfer in these habitats, and the community-level diversity patterns of bacteria carrying these genes. As development accelerates in the Arctic, freshwater systems are at a greater risk of being becoming contaminated by local human activities, and it is unclear how this will impact their role as reservoirs for ARGs. It is therefore critical to improve our understanding of ARG distribution in the North.

## Materials and methods

### Study sites and sampling

**Figure 1.**
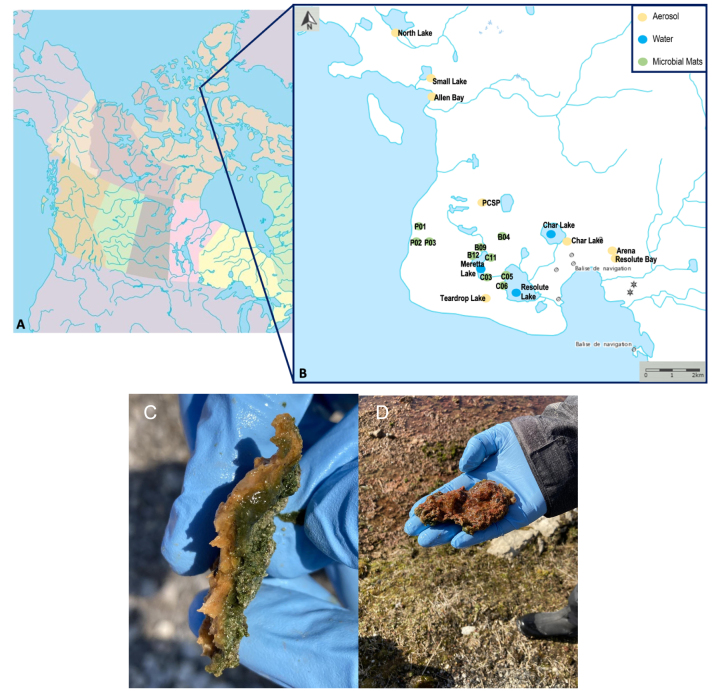
(A) Location of Cornwallis Island, Nunavut, Canada. (B) Map of southern Cornwallis Island. Sampling sites are indicated with colored dots (aerosols in yellow, lake water in blue, and microbial mats in green). Map modified from the Government of Canada (2021). (C) and (D) Microbial Mats collected on Cornwallis Island.

Our study sites are located on Cornwallis Island (75.105°N, 94.828°W) in the Canadian Arctic Archipelago (Fig. 1A). The hamlet of Resolute Bay (Qausuittuq; approximately 180 residents; Statistics Canada 2021), a Canadian federal government hub (Polar Continental Shelf Program; PCSP, Natural Resources Canada) and a small airport approximately 5 km from the hamlet, are located on the southern coast of the island (Fig. 1B). Although remote, the island has been impacted by significant anthropogenic activity during the 20th century. From 1949 to 1998, a military base was operated on the island, which discharged its wastewater directly into the Meretta Lake watershed (Antoniades et al. 2011), accumulating in the lake and its adjacent wetlands (Fig. 1B) (Stewart et al. 2014). This led to a decrease in the water quality of Meretta Lake, making it an uncommon example of a eutrophic High Arctic lake (Schindler et al. 1974, Antoniades et al. 2011). Indeed, Arctic lakes are mostly oligotrophic and examples of eutrophication at the poles are rarely documented (Ayala-Borda et al. 2021).

The southern coast of Cornwallis Island also includes two other nearby waterbodies, Char Lake and Resolute Lake (Fig. 1B). Char Lake is an oligotrophic lake that is the source of drinking water for the hamlet (Michelutti et al. 2003), which is not connected to Meretta Lake and retained its low nutrient concentrations during the sewage discharge era (Schindler et al. 1974). Resolute Lake, a popular fishing site for community members, is located downstream from Meretta Lake, to which it is connected via an approximately 1 km-long stream. These three lakes are therefore located along a gradient of anthropogenic influence, with Meretta Lake and its associated wetlands being most impacted (hereafter referred to as high impact), Resolute Lake experiencing a lower potential impact from its connection to Meretta (mid impact), and Char Lake at the lowest impact point in the gradient (low impact).

**Figure 2.**
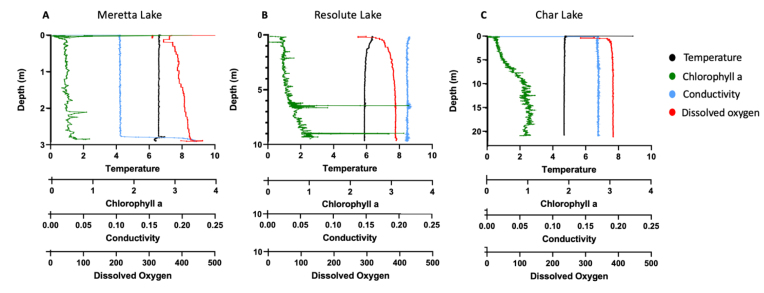
Physicochemical profiles of (A) Meretta Lake, (B) Resolute Lake, and (C) Char Lake. Temperature is measured in °C, chlorophyll *a* concentrations in µg/l, specific conductivity in mS/cm and dissolved oxygen in µmol/l.

Samples were collected in July 2018, August 2019, and August 2021 (63 samples in total). In 2018, aerosols were collected with a SASS4100 two-stage aerosol collector (Research International, Monroe, WA, USA) around the lakes of southern Cornwallis Island (Fig. 1B). Aerosols were collected in duplicate over the course of 1 h at a flowrate of 4000 l/min onto electret filters and then stored at −80°C until processing at Université Laval. Meteorological conditions were obtained from the Environment Canada weather station on Cornwallis Island (Supplementary Table S1). In 2019, the water columns of Meretta, Resolute, and Char lakes were sampled in triplicate at multiple depths (Meretta: 2 and 6 m, Resolute: 2, 10, and 15 m, and Char: 2, 7, and 15 m), with a Limnos water sampler (Limnos.pl, Komorów, Poland). Water was stored in high-density polyethylene Cubitainers, which were pre-washed with 2% vol/vol Contrad^TM^ 70 liquid detergent (DeconLabs, King of Prussia, PA, USA), 10% vol/vol ACS-grade HCl (Sigma–Aldrich, St. Louis, MO, USA), rinsed 3 times with distilled water, and rinsed 3 times with lake water prior to sample collection. Water was then concentrated onto 0.02 µm Anotop™ filters (Whatman, Maidstone, UK) in the laboratory within 6 h of sampling, which were stored at −80°C. Volumes processed are reported in Supplementary Fig. S2. Physicochemical parameters in the lakes were measured with a RBRconcerto CTD + profiler (RBR Global, Kanata, Canada) (Fig. 2). In 2021, 10 microbial mats were retrieved in triplicate from different sites throughout the wetland around Meretta Lake and near the Arctic Ocean (Fig. 1B). In 2021, when microbial mats were sampled, it was not possible to visit Char Lake—mats from a different area of limited anthropogenic impact (P01, P02, and P03) approximately 4 km to the east of Char Lake were included into the low impact area. All mats were collected with sterile spoons and placed in sterile Whirl-Pak™ bags (Filtration Group, Austin, TX, USA). Water temperature, conductivity, pH, and oxygen concentrations were also measured at the wetland sites using the RBRconcerto probe and are reported in Supplementary Table S3. In addition to the molecular analyses described below, pigments were extracted from mat samples in triplicate 5 × 5 mm subsamples into a final volume of 4 ml 90% acetone: water (vol/vol) in the dark and on ice as in Bonilla et al. 2009. Pigments were identified and quantified in each sample based on high-performance liquid chromatography (HPLC) using the protocol described in Zapata et al. (2000). Concentrations of pigments such as *β,β*-carotene, scytonemin, fucoxanthin, and zeaxanthin were determined to further characterize microbial mat samples, and are presented in Supplementary Table S4.

### Nucleic acid extraction and processing

Aerosols were extracted from electret filters using a SASS3010 particle extractor (Research International, Monroe, WA, USA) following Mbareche et al. (2019) and a 0.1 M sodium phosphate buffer solution (pH 7.4), from which nucleic acids were isolated with the QIAamp Viral RNA Mini kit (QIAGEN, Hilden, Germany) following the manufacturer's instructions. Nucleic acids were extracted from Anotop filters following the backflushing method (Mueller et al. 2014) with the MasterPure^TM^ Complete DNA and RNA purification kit (Lucigen, Middleton, WI, USA). Nucleic acids from microbial mats were extracted with the DNA, RNA, and Protein Extraction from Soil Samples Kit following the manufacturer’s instructions (Macherey-Nagel, Düren, Germany). Masses of microbial mats are reported in Supplementary Table S5. Nucleic acids extracted from all sample types were quantified with a Qubit 4 fluorometer using the Quant-iT dsDNA High-Sensitivity Assay Kit (Thermo Fisher, Waltham, MA, USA) and stored at −80°C until sequencing. Nucleic acid concentrations are presented in Supplementary Table S6.

Metagenomic libraries were produced from all sample types with the Nextera Flex kit (Illumina, San Diego, CA, USA), and paired-end sequencing was performed on an Illumina NextSeq at the Integrated Microbiome Resource facility (Dalhousie University, Halifax, Canada). Due to low nucleic acid yields from aerosol samples, DNA from this sample matrix was amplified prior to library preparation with the Accel-NGS 1S Plus DNA library kit (Swift Biosciences, Ann Arbor, MI, USA). Sequencing produced a total of 1 106 456 reads for aerosol samples, 221 516 391 reads for lake water and 179 670 055 reads for microbial mats.

### Sequence analyses

Quality control was performed on NextSeq reads, with low-quality regions removed with Trimmomatic (v.0.36) (Bolger et al. 2014) using a sliding window size of 4, a sliding window minimum quality of 15, and removal of unpaired reads. Read quality was inspected using FastQC (v.0.11.5) (Andrews 2010).

Quality-filtered reads were then parsed to detect ARGs. We used the MEGARes (v.2.0.0) database to identify ARGs in our data (Lakin et al. 2017) and the AMRplusplus (v.2.0) pipeline to compare these sequences to those in the MEGARes database (v.2.0.0) (Doster et al. 2020). Quality-controlled reads were aligned to the MEGARes database using the Burrows–Wheeler–Aligner (v.0.7.17) (Li et al. 2009) to determine the presence of ARGs across individual samples.

Metagenomic reads were also assembled to characterize bacterial and viral genomes carrying ARGs. Quality-controlled reads from each sample were assembled *de novo* with MetaSPAdes (v.3.13.0) with settings as described in Nurk et al. (2017). This produced a total of 2 contigs for aerosol samples, 236 570 contigs from lake water and 89 589 contigs from mats. All contigs below 10 000 bp in length were removed (Song 2021), resulting in 14 847 lake water and 4320 mat contigs for subsequent diversity analyses. Both contigs assembled from aerosol samples were below this cut-off and were therefore not retained for downstream bacterial and viral diversity analyses. Viral contigs were identified within the lake water and microbial mat assemblies with VirSorter2 (v.2.2.1) (Guo et al. 2021), and VPF-Class (v.0.1.00) (Pons et al. 2021), resulting in 7874 and 55 viral contigs in water and mats respectively. To determine if these contigs carried ARGs, we parsed viral-identified contigs using BLASTn (v.2.9.0) (National Center for Biotechnology Information 2022) and the MEGARes database (v.2.0). Only hits that had an identity percentage >85% and an *e*-value <0.05 were retained.

Finally, we performed a principal coordinate analysis (PCoA) with Bray–Curtis dissimilarity to visualize the differences in community assemblages amongst sample types and across high, mid, and low-impact sites. Briefly, raw reads were classified with Kraken2 (v.2.0.8-beta) (Wood et al. 2019), and biom tables were generated from this output with kraken-biom (v.1.2.0) (Dabdoud 2016), which were then converted to operational taxonomic units (OTUs) tables for visualizations in PCoA space. These data were imported into R (v.4.3.3) (R Core Team 2023) and processed with the phyloseq (v1.46.0) (McMurdie et al. 2013), ggplot2 (v.3.5.0) (Wickham 2016), RColorBrewer (v1.1.3) (Neuwirth 2022) and patchwork (v.1.2.0) (Pedersen 2024) packages to analyze the bacterial diversity and community patterns across habitat type (aerosols, lake water, and mats) and level of impact (high, mid and low). Analysis of variance (PERMANOVA) comparing habitats and anthropogenic impact was calculated with the adonis() function, and group dispersion homogeneity was verified with betadisper() from the vegan (v.2.6.4) package (Oksanen et al. 2022). ARG distributions across samples were also plotted as vectors over a PCoA from log-transformed data and a Bray–Curtis dissimilarity matrix in Primer 7 (v7.0.21) (Clarke et al. 2015). Measures of alpha diversity (Observed richness, Chao1, Shannon index) and ARG family diversity were compared using a Kruskal–Wallis rank sum test in the stats package (R Core Team 2023) followed by Dunn’s post hoc with Holm correction with the package FSA (v.0.9.5) (Ogle et al. 2023), as normality (checked with shapiro.test() in the stats package) was not respected.

## Results

### Environmental conditions of aerosol, lake and microbial mats

The Cornwallis Island landscape is polar desert, characterized by sparse vegetation and a cold dry climate. During aerosol sampling in 2018, the temperature was below 10°C. The lowest temperature was 0.7°C at the PCSP site (Supplementary Table S1). Relative humidity was highest at PCSP, teardrop, and small lake (98%, 91%, and 89%, respectively) and the highest wind speeds (up to 41 km/h) were measured during sampling at the Char Lake site (Supplementary Table S1).

During lake water sampling in 2019, Meretta Lake had a cold (6°C), unstratified water column (Fig. 2A). Oxygen increased slightly towards the bottom (462 μmol/l), and this lake had the lowest conductivity values of any of the sampled lakes (0.10 mS/cm). Downstream of Meretta Lake, Resolute Lake had temperature and conductivity values that also remained constant throughout the water column, with values of approximately 6°C and 0.20 mS/cm, respectively (Fig. 2B). Chlorophyll *a* concentrations increased with depth from 0.02 µg/l to 3.41 µg/l. Char Lake is a cold, ultra-oligotrophic monomictic lake, and during sampling, temperature and conductivity in Char Lake remained constant at approximately 5°C and 0.17 mS/cm throughout the water column, and chlorophyll *a* concentrations were below 1.0 µg/l at all depths (Fig. 2C).

For microbial mats sampled in 2021, physicochemical measurements revealed higher temperatures in mats in shallow water (P03, B04, and B09) (Supplementary Table S3). Among these, site P03 had relatively more black microbial mats, which may have resulted in more heat absorption and higher temperatures, while B04 was dominated by dense green algae. Site B09 was adjacent to one of Meretta Lake’s inflows. The pH was constant at all mat sites, ranging from 8.11 (C06) to 8.62 (B12) (Supplementary Table S3). At most sites, the UV-screening pigment scytonemin dominated (Supplementary Table S4), with highest scytonemin ratios in the P03 mat. Chlorophyll *a* concentrations were greater in the B12 and P01 samples, which were orange on the surface with a blue–green layer underneath (Fig. 1C and D).

### ARGs detected across habitat types throughout the anthropogenic gradient

We detected a total of 984 ARGs across all samples, which grouped into 17 resistance genes (Supplementary Table S7) belonging to 9 ARG families (Table 1). We did not detect ARGs against β-lactam, trimethoprim, and vancomycin in any of the samples. ARGs were found in high, mid and low-impact sites, and high-impact sites had significantly more families detected than low-impact ones (*P* < 0.05). A16S (resistance to aminoglycosides), MLS23S (resistance to MLS) and *tufAB* (resistance to elfamycin) were the most prevalent (Supplementary Table S7). Microbial mats contained the greatest relative abundance of ARGs, with 21.67% of contigs >10 000 bp carrying resistance genes; however, ARGs were detected in only one aerosol sample, which had the lowest ARG diversity (*P* < 0.05, Table 1). This sample was collected in the hamlet and contained genes encoding resistance to aminoglycosides and tetracyclines (A16S and TET16S). The presence of ARGs correlated with mat and lake water communities, and negatively with aerosols (with the exception of the hamlet sample) (Supplementary Fig. S1). Although aminocoumarins, cationic peptides and *Mycobacterium tuberculosis* drug resistance genes were found in mats, they were absent from lake water (Table 1). Both water column and mat samples contained genes associated with resistance to aminoglycosides, fluoroquinolones, MLS (macrolides, lincosamides, and streptogramines), rifampins, tetracyclins, and elfamycin. Aminoglycosides and MLS resistance were the most detected families and were common to all three lakes. Meretta Lake contained the fewest number of ARG hits overall (43), while Char Lake had the most (66; Table 1). Aminoglycoside resistance was detected in all microbial mats (A16S being the most prevalent gene), had the greatest diversity of any ARG found in mats (with an average of 44 hits), and was consistently the most diverse in lake water (Table 1).

**Table 1.**
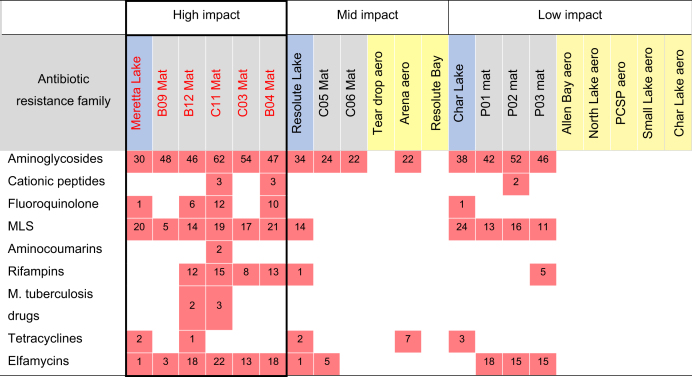
Presence (red) and absence (white) of ARGs found in all sample types according to level of anthropogenic impact.

Microbial mat samples are in green, aerosol samples in yellow and lake samples in blue. Names of sites in the high impact area (Meretta Lake and its surroundings) are labelled in red. Numbers in the boxes indicate the average number of hits found with the MEGARes database for each antibiotic family in the sample.

We compared the distribution of ARGs detected in the high impact (Meretta Lake and surroundings) to the mid and low-impact sites. The aminoglycoside resistance gene A16S and elfamycin-resistance *tufAB* were detected in all high impact samples, and many genes were only detected in the impacted area (*rrsC*, AAC(3), AAC(6′) (Supplementary Table S7). High impact sites around Meretta Lake had a greater diversity of ARGs, with a total of 9 families detected, while the mid and low impact had 5 and 7 families detected, respectively (Table 1). Antibiotic resistance families such as aminocoumarins and *M. tuberculosis* drug resistance were restricted to mats in the high impact area. Resistance to aminoglycosides was found in all sites across the anthropogenic gradient, from high impact sites to low-impact areas, and including in the ARG-containing aerosol sample, but ARGs relevant to this antibiotic family were most diverse in the high impact area. Resistance to tetracyclines was present at low abundances in high, mid and low-impact sites in all habitats types. Mats in the high-impact area around Meretta Lake contained the most hits to ARG families (Table 1). Microbial mats in the low-impact area (P02, P03), located further away from wastewater discharges and closer to the Arctic Ocean, contained ARGs encoding resistance to aminoglycosides, cationic peptides, MLS, rifampins, and elfamycins.

The most diverse sample across the entire dataset in terms of number of ARGs was microbial mat C11 (in which all resistance-encoding genes except TET16S were detected), located in the high impact area near Meretta Lake. It is also the only site where resistance to the aminocoumarin family of antibiotics was detected. Conversely, mat site B09 had the lowest diversity of resistance in the high impact area around Meretta.

**Figure 3.**
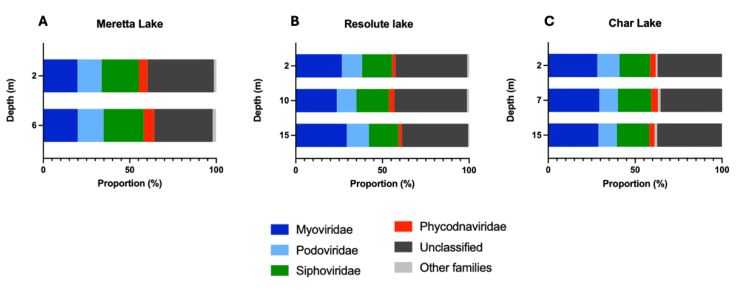
Relative abundance of viral families in (A) Meretta, (B) Resolute, and (C) Char Lake. Families accounting for <2% relative abundance were grouped as “Other families”.

To better understand the role of viruses as potential drivers of ARG distribution in this environment, we identified the type of genome (bacterial, viral) carrying the ARGs we detected in our metagenomic sequences. Viral contigs comprised 0.7% of the total contigs, and of these, four included an identifiable ARG, which were all from lake water (Table 2). All other ARGs were carried on non-viral contigs. We characterized the viral community in the lake waters where viral-carried ARGs were detected. The three lakes were dominated by viruses in the families *Myoviridae, Podoviridae*, and *Siphoviridae* (Fig. 3). A substantial proportion of viral contigs could not be classified at the family level (Fig. 3).

**Table 2.** ARGs in viral contigs detected in lake water at different depths and replicates (A, B, and C).

Sample	Family annotation of viral contig	ARG family
Meretta Lake 6mA	*Mimiviridae*	MLS resistance
Meretta Lake 6mC	*Mimiviridae*	MLS resistance
Resolute Lake 2mB	Unknown	Major common ARG
Resolute Lake 15mC	*Myoviridae*	Rifampicine resistance

### Microbial community composition

**Figure 4.**
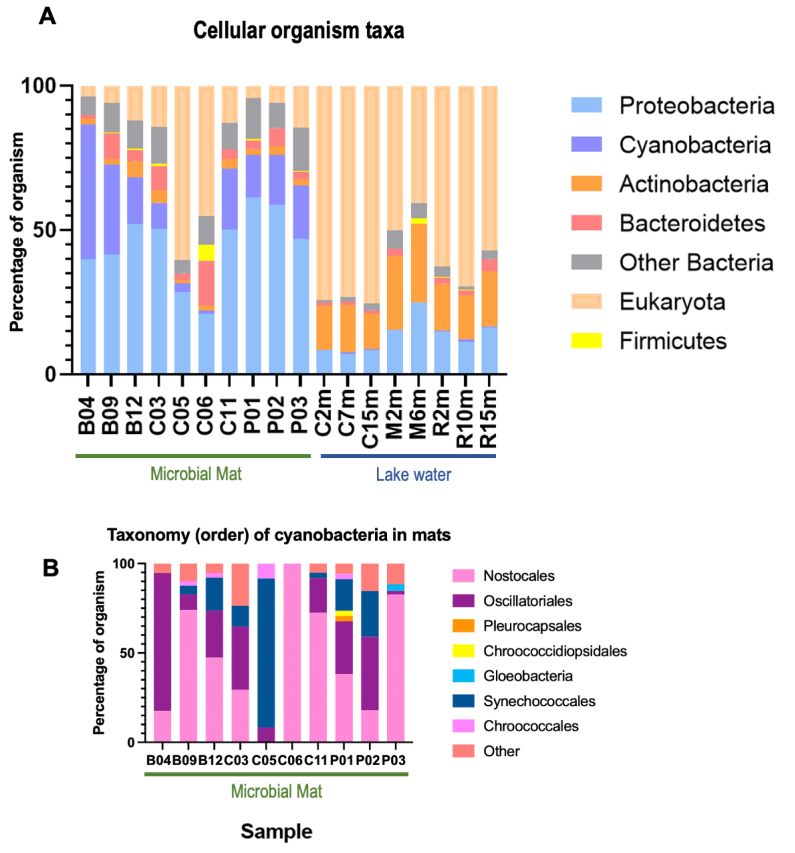
Microbial community composition of lake water and microbial mat samples. (A) Phyla present in microbial mat and lake water samples. (B) Cyanobacterial orders in mat samples.

Most contigs in the assembly belonged to cellular organisms, and microbial mats had the greatest prokaryotic and eukaryotic diversity. Mats in the high (B04, B09, B12, C03, and C11) and low-impact (P01, P02, and P03) areas were dominated by bacteria (especially Proteobacteria and Cyanobacteria), while mats in the mid-impact area (C05, C06) were enriched in Eukaryotes (Fig. 4A). Lake water samples were dominated by Eukaryotes and Actinobacteria (Fig. 4A). For bacteria-dominated mats, we explored the cyanobacterial diversity of these sites, and found that Nostocales were the most abundant group, with Oscillatoriales and Synechococcales also well represented (Fig. 4B). No aerosol contigs >10 000 bp remained following quality-control of the assembly, therefore no aerosol diversity data are presented here.

**Figure 5.**
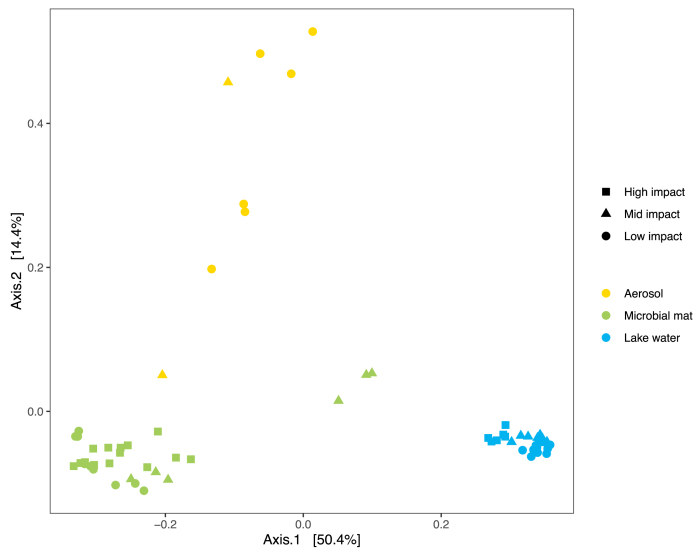
Bacterial PCoA from Bray–Curtis dissimilarity by habitat for lake water (blue), microbial mats (green), and aerosols (yellow) of impacted and non-impacted sites. Squares represent high impact sites, while triangles represent mid impact and circles low-impact sites.

Bacterial communities clustered broadly by habitat type (*R*^2^ = 40.9%, *P* < 0.001) (Fig. 5), although there was significant dispersion among groups (*P* < 0.001, 999 permutations). Anthropogenic impact was a weaker, yet significant, driver of community assemblages across all habitat types (*R*^2^ = 9.8%, *P* < 0.01). Biodiversity measures were generally significantly greater in mats and water (Chao1 index, *P* < 0.0001), and among these, mats had greater species evenness, with lake water yielding significantly lower Shannon index values (*P* < 0.001). However, observed richness was not significantly different across levels of impact (*P* > 0.05).

## Discussion

### Contrasting environmental conditions across the anthropogenic impact gradient

We found a strong gradient in environmental parameters when comparing the three lakes across the anthropogenic gradient. Although Meretta Lake was eutrophic in the past, with chlorophyll *a* concentration ranging from 3 to 5 µg/l in the 1970s (Douglas et al. 2000, Antoniades et al. 2011), these concentrations have now returned to pre-contamination levels below 0.9 µg/l. While temperature (6°C in 1992 vs. 0°C in 2019) and conductivity (0.10 mS/cm vs. 0.094 mS/cm) were higher in 1992, chlorophyll *a* concentrations were lower (0.9 µg/l vs. 2.1 µg/l) (Douglas et al. 2000). These trends suggest that Meretta Lake is in the process of recovery after the stopping of sewage inputs (Wu et al. 2020). Chlorophyll *a* levels in Resolute Lake were higher than those measured prior to 2002 (Campbell et al. 2005), but consistent with data collected from 2005 to 2016 (Cabrerizo et al. 2018). Conductivity measurements were slightly lower than those measured in Resolute Lake from 1997 and 2002 of approximately 0.252 ± 0.033 mS/cm (Campbell et al. 2005). As conductivity is a good indicator of water condition and can be used to determine the impact of anthropogenic activity on lake water (Wu et al. 2020), Resolute Lake appears to have remained limnologically stable over time, despite sewage inputs into nearby Meretta Lake. In Char Lake, our values are consistent with observations made in prior to 2000 (Michelutti et al. 2003), during which summer temperatures rose from 3.5 to 7.0°C and chlorophyll *a* concentrations were <0.1 µg/l, suggestion stable conditions in Char Lake of the past decades, consistent with its isolation from Meretta Lake. These three lakes therefore exhibited the anthropogenic impact gradient of high, mid to low that we expected.

As expected, we found that the distinctly different conditions of aerosols, lake water, and mats would shape different bacterial communities, as has been observed in other studies on bioaerosols (Péguilhan et al. 2023), Arctic ice shelf microbial mats (Bottos et al. 2008) and Arctic freshwaters (Crump et al. 2012). While impact explained a limited proportion of bacterial assemblages (and did not impact species richness), the stonger clustering of of communities by habitat, especially for lake water, suggests important selective pressures for aquatic organisms and more homogenous freshwater communities across the landscape despite anthropogenic impact (Fig. 5). The pattern is much less evident in mats (Fig. 5), where there is no clear grouping of communities by anthropogenic impact and a pronounced heterogeneity in community composition among sites. Microbial mats are known to be highly heterogeneous at both micro and macro size scales (see Supplementary Discussion), as well as over time; sampling microbial mats at discrete locations and as single points in time therefore provides only snapshots of their composition (Bolhuis et al. 2014). The aerosol samples showed the greatest heterogeneity and lowest values of alpha diversity, consistent with the displacement of air masses across the land and sea, with variable exchanges of microbiota.

Our expectation was that microbial communities would cluster together based on degree of impact as found by Elo et al. (2018), who showed that changes in the chemical properties of lakes due to eutrophication impacted bacterial species richness, with higher values of pH, alkalinity, and conductivity resulting in distinct and richer species diversity. In this dataset, habitat type seems to be a stronger driver of contemporaneous bacterial diversity on southern Cornwallis Island, with a limited signal from historical exposure to wastewater.

### ARG diversity in High Arctic watersheds

Our survey of these High Arctic sites revealed the presence of multiple ARGs, despite the area being far from large urban centers. This may be attributable to anthropogenic impacts of sewage inputs during the 20th century, modern use in the nearby community and long range transport, as well as naturally occuring resistance. We detected ARGs encoding resistance to 9 antibiotic families: aminoglycosides, cationic peptides, fluoroquinolone, MLS, aminocoumarins, rifampins, *M. tuberculosis* drugs, tetracyclines, and elfamycins. A previous study in Antarctica tested 15 clinically used antibiotics on 229 bacterial strains from Antarctic soil samples (Marcoleta et al. 2022). Among these strains, resistance was observed for 13 of these 15 antibiotics, suggesting widespread resistance despite the remoteness of these sites. Interestingly, this included multiple ARGs that were not detected in our study (resistance to tocolistin, clindamycin, fosfomycin, trometamol, etc.). ARGs relevant to aminoglycosides (A16S, *rpsL, rrsA, rrsC, rrsH*) and elfamycins (*tufAB*) were widely distributed across our sites. In contrast, resistance to cationic peptides, *M. tuberculosis* drugs and aminocoumarins were quite rare (3 samples or less), and were only found in microbial mats. A study of pristine soils in Antarctica found resistance to aminocoumarins in only 7 of the 17 sites studied (Van Goethem et al. 2018), supporting our observation that aminocoumarin resistance is uncommon in remote environments.

The absence of ARGs against β-lactam, trimethoprim, and vancomycin in our samples was unexpected as these types of genes have been identified in wastewater (Zhang et al. 2009) and β-lactam ARGs have also been found in soils from natural environments as well as surface and groundwater (Graham et al. 2016). However, our ARG identification approach was limited to searches against the MEGARes database which does not include the NCBI Lahey Clinic beta-lactamase archive (Doster et al. 2020). The first clinical use of trimethoprim (diaminopyrimidines) as a first-line antibiotic was in 1968 (Eliopoulos and Huovinen 2001). Even though this was during the height of military activity on Cornwallis Island, the drug was still relatively new and unlikely to have been used in high quantities, which may explain why we did not find ARGs to this antibiotic.

### Ubiquity of ARGs across habitats

All habitats we surveyed harboured genes encoding antibiotic resistance. While they were less prevalent and less diverse in aerosol samples (with only one sample generating hits), all three lakes, and all but one microbial mat contained ARGs. As shown in DNA yields prior to amplification, aerosol samples contain low biomass (Luhung et al. 2015), potentially explaining the fewer ARGs we found in this habitat. This is similar to the low microbial biomasses and ARG detection limits found in a study of atmospheric deposition in a remote mountain area in Italy, where resistance genes associated with tretracycline and sulfonamide were detected (Cáliz et al. 2022). We found genes conferring resistance to aminoglycosides (A16S) and tetracycline (TET16S) in one aerosol sample near the hamlet, which may point to influence from the nearby marine habitat. The Arctic Ocean is known to be a source of bioaerosols (Wéry et al. 2018, Hartmann et al. 2020), and open water can be a source of bioaerosols to areas where their local sources are otherwise limited. The detection of ARGs in this sample may therefore be connected to its proximity to the ocean.

The ARG profiles in Meretta, Resolute, and Char lakes were similar, with comparable numbers of hits to genes encoding resistance to aminoglycosides (A16S, *rrsA*), MLS (MLS23S), and tetracyclins (TET16S). These results are consistent with Jara et al. (2020), who investigated antibiotic resistance in freshwater samples from Antactica. Among their samples from three lakes differentially impacted by anthropogenic activities, they found, as did we, resistance to aminoglycosides and tetracycline. They also found resistance to β-lactams, as well as to ciprofloxacin, chloramphenicol and sulfamethoxazole (Jara et al. 2020), which we did not detect in the Cornwallis Island lakes.

Microbial mats had the greatest ARG diversity across all habitats (Table 1): all mats but one contained ARGs relevant to multiple antibiotic families, and one mat (C11) contained 16 of the 17 resistance-encoding genes detected in this study. The greater ARG diversity in mats may be due to longer residence times are greater in these biofilms compared to aerosols and lake water, due to relatively small advective forces (Prieto-Barajas et al. 2018), allowing for longer retention time and potential concentration of ARGs in this habitat. Mats also have much higher density of cells than lake water and aerosols (Prieto-Barajas et al. 2018) which could favour greater diversity of resistance mechanisms through more complex ecological interactions. DNA in mats may also be better protected from degradation, moreso than in bioaerosols, where low biomass leaves cells more vulnerable to degradation (Luhung et al. 2015), or even compared to lake water, where temperature can affect the degradation of DNA (Eichmiller et al. 2016). Finally, DNA protection from UV degradation by the high concentrations of photoprotective pigments in the mats may have contributed to the greater diversity of ARGs we found in this habitat type, as UV radiation is used as a treatment against resistance in wastewater plants (Manoharan et al. 2022).

### High anthropogenic impact areas had greater ARG diversity

Little is known on the distribution of ARGs in polar regions. This is a pressing area of research, as global warming and increased human activity are expected to mobilize ARGs further North. Our study area offers a unique opportunity to compare the diversity and distribution of ARGs of anthropogenic (from historical sewage inputs into Meretta Lake) and naturally occurring (in mid and lower impact sites) origins. Our limnological data show that Meretta Lake has since recovered from its eutrophication, but an ARG signal may have persisted since sewage disposal ceased in 1998, and as expected, greater ARG diversity was observed in the high impact sites. Here, we found the greatest diversity in ARG families, which is probably a legacy effect of receiving untreated sewage from 1949 to 1998, which likely contained unprocessed antibiotics and resistant bacteria (Mtetwa et al. 2021). Such long-term direct exposure to raw sewage possibly led to the persistence of resistance in the microbial communities of the Meretta area, as demonstrated by the high number of ARGs we detected over two decades after the end of the exposure period. The high ARG diversity we found in mats around Meretta Lake is likely due to their proximity to the aqueduct used to transport wastewater to the lake, and mats closer to to the discharge (C11) retaining greater numbers of ARGs, while those located near an older, previously discontinued wastewater canal (B09) experienced a shorter exposure time and has had a longer time to recover, leading to fewer ARGs. This may be an underestimate the true diversity of ARGs in this area, given the identity threshold we used to match against databases (85%), meaning ARGs below this threshold may not have been captured. Still, our study provides a first snapshot of modern ARGs around Meretta Lake following the sewage inputs of the 20th century.

We also found ARGs in the mid to low impact sampling sites. These likely have natural origins, as these sites exhibit limited or no hydrological connectivity with Meretta. While aerosols may be a potential vector for ARG dispersal (Ling et al. 2013), this does not appear to be the primary vector in our sites, given the low detection of ARGs in bioaerosols. Contrary to our expectations, all three lakes had comparable ARG profiles. This may be attributable to the fact that in-lake selective pressures dominate microbial assemblages and the selection of ARGs in the water column after sewage disposal ceased over two decades ago, with subsequent flushing of these waters by snow melt each year. To further investigate this, deep sequencing of sediment from Meretta, Resolute, and Char lakes could provide further information of historical loading of ARGs during the wasterwater discharge period.

### Potential origin of ARGs

As microbial mats may act as long-term reservoirs for ARGs, resistance-encoding genes found only in mats around highly impacted Meretta Lake may be of anthropogenic origin [*rrsC*, AAC(3), AAC(6′), *pare, rpsA, thyA*]. Meanwhile, ARGs that were detected in the mats in the mid and low impact sites (A16S, MLS23S, *tufAB*) were all also found in high impact mats: these genes likely represent those naturally present in uncontaminated Arctic environments. Placing these naturally-occurring ARGs into their wider Arctic context is difficult, as few studies have focused on this topic. A study of antibiotic sensitivity present in the mucosa of Arctic fish showed resistance to ampicillin, chloramphenicol, kanamycin, streptomycin and tetracycline. The fishing site was at least 120 km from the closest village (Moniz et al. 2022) demonstrating that ARGs occur naturally in remote Arctic environments. It is also possible that these ARGs may have been introduced from impacted areas by migratory birds (Moniz et al. 2022), as has been observed in Antarctica (Segawa et al. 2024), and are therefore unrelated to past sewage inputs.

The presence of aminoglycoside resistance (A16S) across the entire impact gradient supports observations from previous studies showing that aminoglycoside resistance is common across diverse environments such as groundwater, seawater, soil and fish microbiomes (McCann et al. 2019, Chen et al. 2020, Felis et al. 2020, Jia et al. 2022). Aminoglycoside resistance has been found in Antarctica in wastewater from a research station (Hwengwere et al. 2022) and in both polluted as well as natural water environments (Zhang et al. 2009) in the vicinity of McMurdo Station. The pervasiveness of aminoglycoside resistance genes in our samples may be due to the fact that aminoglycosides were among the first antibiotic families used to treat bacterial infections in the 1940s and have thus been circulating for a relatively long time in the environment (Krause et al. 2016). Indeed, once they are present in the environment, ARGs are readily transferred by horizontal gene transfer and become difficult to eliminate (Su et al. 2020), increasing the likelihood of detecting older resistance mecanisms to older antibiotics. However, aminoglycosides are also naturally produced by soil microorganisms such as Actinobacteria (Coates et al. 2022), which we also found in microbial mats (Fig. 4A). Furthermore, there is also evidence of their natural occurence at high latitudes: a study of a 5000-year-old Arctic permafrost core revealed the presence of aminoglycoside resistance mechanisms in the Arctic long before the use of clinical antibiotics (Perron et al. 2015), supporting the natural backround signal of resistance to this antibiotic family on Cornwallis Island.

Resistance to tetracycline, another major antibiotic used since its discovery in the 1940s, was also found across the gradient (encoded by *tufAB*), suggesting a natural origin, independent of sewage inputs into Meretta Lake. Tetracyclines are known to be omnipresent and resistant to degradation in some environments (Daghrir et al. 2013, Shao et al. 2020). Further, tetracycline compounds are common in soils (Thaker et al. 2010). One study has shown that resistance to tetracycline antibiotics is found in all types of water samples in North America including in surface water and in lagoons (Zhang et al. 2009). While this evidence comes from studies of these ARGs in temperate, densely populated areas with multiple anthropogenic influences, it shows that tetracycline resistance in the environment is pervasive. Resistance to tetracycline has previously been detected in permafrost cores collected in the High Arctic (Perron et al. 2015), supporting the idea that tetracycline in our gradient may be unrelated to modern sewage inputs in Meretta Lake.

### Viruses are not the main vectors of ARG dispersal

Bacteriophages may play an important role in the dissemination of ARGs in bacteria (Debroas et al. 2019, Chen et al. 2021). Although our viral contigs demonstrate that ARGs were detectable in this fraction of metagenomic data, they give limited insight into the role of viruses in the dissemination of ARGs. This is most likely due to undersampling of the Cornwallis Island virome, and to the lack of polar viruses in current sequence databases. Other authors have also found that viral-associated ARGs are rare (Enault et al. 2017), including in polar habitats (Liu et al. 2023). Nonetheless, it is interesting that the MLS resistance genes identified in two Meretta Lake samples were associated with the family *Mimiviridae*. Mimiviruses are a type of giant virus that infects amoebae and other protists, with large genomes that can exceed 1.2 Mbp in length and contain over 900 genes (Colson et al. 2017, Claverie et al. 2018), and have been recently detected in Arctic lakes (Pitot et al. 2024). Even though ARGs conferring resistance to several antibiotics (β-lactam, macrolide, metronidazole, and tetracyclines) have been found in the mobilome of samples containing giant viruses (Chatterjee et al. 2019), to the best of our knowledge, this is the first observation of an ARG present in the potential genome of a giant virus.

## Conclusion

We detected ARGs in all habitats surveyed in this study, including aerosols, lake water, and microbial mats. Resistance to the aminoglycoside family was the dominant form of resistance observed across all habitat types. Aerosols contained fewer ARGs, likely due to the lower amounts of DNA in these samples. Lake water and microbial mat samples contained numerous resistance genes, including resistance to aminoglycosides, aminocoumarins and tetracyclines. Microbial mats in the high impact area had the highest diversity of ARGs relative to mid and low-impact sites, but this was not the case for the lake waters and aerosols. While some ARGs were found across the impact gradient (suggesting a natural origin), others were restricted to mats around Meretta Lake, suggesting long-term consequences of sewage inputs into the lake in the 20th century. This may be due to several factors that preserve the signal of contamination in the form of antibiotic resistance in mats. More research is needed to understand the complex ecological interactions of mat communities and the ways in which resistance is maintained in this habitat. There was a small yet significant relationship between bacterial community composition and contamination, but community composition was mostly driven by habitat type. ARGs were predominantly observed in cells and not in viruses. Nonetheless, our data suggests that some giant viruses can harbour ARGs. This study is important because anthropogenic activities are likely to increase in the polar regions as a result of global warming, and monitoring the emergence of resistance in the environment in real time could avert certain problems for human health. Also, as we have detected the presence of ARGs in Mimivirus genomes, it would be interesting to deepen this research to understand why a virus that presumably infects a eukaryote possesses an ARG.

## Supplementary Material

qvae021_Supplemental_File

## Data Availability

Raw sequencing reads are deposited on NCBI in BioProject PRJNA1101925 at https://www.ncbi.nlm.nih.gov/bioproject/.
