# Supplementary material for: Microbial antibiotic resistance genes across an anthropogenic gradient in a Canadian High Arctic watershed
Source: Sustain Microbiol. 2024 Aug 7;1(1):qvae021. doi: 10.1093/sumbio/qvae021 (PMC13409144; doi:10.1093/sumbio/qvae021)

Supplementary material: Microbial antibiotic resistance genes across an anthropogenic gradient in a Canadian High Arctic watershed

Juliette Provencher[1,2,3,4], Paul B.L. George[1,2,5*] Mary Thaler[1,2,3,4], Warwick F. Vincent[2,3,4,6], Caroline Duchaine[1,5], Alexander I. Culley[1,2,3,4†], Catherine Girard[2,3,7*]

1. Département de biochimie, de microbiologie et de bio-informatique, Université Laval, Québec, QC, Canada
2. Centre d'études nordiques (CEN), Université Laval, Québec, QC, Canada
3. Institut de biologie intégrative et des systèmes, Université Laval, QC, Canada
4. Takuvik Joint International Laboratory, Université Laval, QC, Canada
5. Institut universitaire de cardiologie et de pneumologie de Québec (IUCPQ), Université Laval, Québec, QC, Canada
6. Département de biologie, Université Laval, Québec, QC, Canada
7. Département des sciences fondamentales, Université du Québec à Chicoutimi, QC, Canada

† Current affiliation for A.I. Culley: Pacific Biosciences Research Center, University of Hawai'i at Mānoa, Hawai'i, USA

*Corresponding authors: catherine5 _girard@uqac.ca, https://orcid.org/0000-0002-3899-0180; paul.george@bcm.ulaval.ca, https://orcid.org/0000-0002-3835-431X

Supplementary material contains :

- **Supplementary Discussion**
- **Supplementary Tables :**
  - **Supplementary Table S1.** Meteorological data from aerosol sampling events.
  - **Supplementary Table S2.** Lake water volumes processed for DNA extraction.
  - **Supplementary Table S3.** Physicochemical properties of microbial mat sites sampled in August 2021.
  - **Supplementary Table S4.** HPLC pigment ratios for a subset of sites shown in Supplementary Table S1.
  - **Supplementary Table S5.** Microbial mat extraction masses.
  - **Supplementary Table S6.** DNA concentrations of all samples after extraction.
  - **Supplementary Table S7**. Presence (red) and absence (white) of specific antibiotic resistance genes encoding resistance to families reported in Table 1 across samples, degree of impact and habitats.
- **Supplementary Figure :**

- **Supplementary Figure S1.** Principal coordinate analysis of microbial communities by habitat type overlayed with ARG hit vectors using Bray-Curtis dissimilarity matrix.

Supplementary Discussion

Description of microbial mats

Microbial mats were collected throughout the gradient (as shown in Figure 1C & D), and physicochemical and pigment data are presented in Supplementary Tables S3 and S4, respectively. In the high impact area, mat P03 was completely black, which was likely explained by its extremely high scytonemin ratio. Scytonemin is a UV-screening sheath pigment that gives rise to brown or black coloration when in high concentrations (Bonilla et al.. 2005). Other mats near Meretta Lake (B04, C03, C11, B12 and B09) did not share discernable characteristics in contrast to the low-impact sites. Mats at two of these sites (C03 and B12) were orange, while site B04 also contained various green filaments. Site C11 had beige mats including black colonies and B09 contained a black film. Mats therefore showed variability within impact areas.

Supplementary Tables

Supplementary Table S1. Meteorological data from aerosol sampling events.

Site	Sample	Date	Hours	NAVCAN data							
				Time	Temperature (°C)	Dew Point Temp (°C)	Rel Hum (%)	Wind Dir (10s deg)	Wind Dir (deg)	Wind Spd (km/h)	Stn Press (kPa)
Char Lake	A	2018-08-01	14h51-15h41	14:00	4.8	1.0	76	9	90	41	99.43
				15:00	5.5	0.9	72	10	100	33	99.5
	B		15h42-16h32	16:00	5.9	0.8	70	9	90	28	99.51
				17:00	6.2	1.5	72	12	120	23	99.54
Allen Bay	A	2018-08-02	15h55-16h55	15:00	8.8	3.6	70	1	10	20	99.45
				16:00	9.4	4.0	69	36	360	21	99.43
	B		16h56-17h54	17:00	9.7	3.6	66	1	10	17	99.43
				18:00	9.7	4.4	70	2	20	17	99.45
Small Lake	A	2018-08-03	15h28-16h05	15:00	M	M	M	34	340	34	M
				16:00	2.6	1.1	89	32	320	36	99.1
Resolute Bay (Arena)	A	2018-08-04	14h42-15h42	15:00	3.2	1.3	87	11	110	36	99.34
				16:00	3.6	1.6	87	10	100	29	99.34
North Lake	A	2018-08-05	15h52-16h52	15:00	6.3	2.2	75	3	30	23	99.28
				16:00	6.4	2.0	73	4	40	26	99.26
	B		16h52-17h54	17:00	6.7	2.4	74	3	30	23	99.22
				18:00	6.8	2.5	74	4	40	27	99.17
Teardrop	A	2018-08-06	15h25-16h10	15:00	2.1	0.8	91	15	150	11	98.58
				16:00	2.5	0.7	88	16	160	8	98.57
	B		16h10-16h55	17:00	2.0	0.5	90	27	270	11	98.59
PCSP	A	2018-08-07	20h57-22h09	20:00	1.3	1.0	98	24	240	20	99.3
				21:00	1.4	1.1	98	24	240	22	99.29
	B		22h09-23h13	22:00	1.2	0.8	97	23	230	16	99.3
				23:00	0.7	0.3	97	17	170	14	99.31

M = Missing data point

Supplementary Table S2. Lake water volumes processed for DNA extraction.

Lake	Depth (m)	Triplicate	Water volume processed (L)
Char	2	A	0.330
		B	0.345
		C	0.320
	7	A	0.370
		B	0.400
		C	0.395
	15	A	0.340
		B	0.320
		C	0.340
Meretta	2	A	0.165
		B	0.150
		C	0.175
	6	A	0.170
		B	0.170
		C	0.150
Resolute	2	A	0.250
		B	0.310
		C	0.310
	10	A	0.350
		B	0.360
		C	0.350
	15	A	0.330
		B	0.310
		C	0.300

Supplementary Table S3. Physicochemical properties of microbial mat sites sampled in August 2021. Latitude (Lat.), longitude (Long.), water temperature (Temp.), conductivity (Cond.), pH and oxygen are represented.

Site	Lat. (°)	Long. (°)	Temp. (°C)	Cond. (μS/m)	pH	Oxygen (μmol/L)
C03	74.692098	94.977073	6.4	144	8.34	ND
C05	74.690857	94.964351	4.4	169	8.24	ND
C06	74.690662	94.968220	3.9	305	8.11	ND
C11	74.695313	94.991480	6.4	152	8.46	ND
B04	74.707377	94.976456	8.7	324	8.13	12.21
B09	74.699205	94.987014	8.2	251	8.28	11.46
B12	74.696512	95.000927	6.8	144	8.62	12.40
P01	74.706733	95.078075	7.0	196	8.42	10.14
P02	74.704038	95.080216	5.1	277	8.32	10.18
P03	74.704008	95.077758	14.4	157	8.50	11.26

ND not detected.

Supplementary Table S4. HPLC pigment ratios for a subset of sites shown in Supplementary Table S1. All pigment concentrations are in units of chlorophyll *a*, which is considered a proxy for biomass.

	B09	B12	C05	C11	P01	P03
**Concentration (μg/cm^2)**						
Chlorophyll *a*	29.0799	47.4212	9.3988	25.6316	60.0314	13.5914
**Pigment ratios**						
β.β-carotene	0.0706	0.0924	0.0412	0.0935	0.1151	0.0848
Scytonemin	2.9533	0.1240	0.0388	0.5843	0.3028	8.0544
fucoxanthin	0.0000	0.1096	0.1714	0.0000	0.0684	0.0000
zeaxanthin	0.0038	0.0283	0.0860	0.0138	0.0272	0.0033

Supplementary Table S5. Mass of microbial mat samples used for DNA extraction.

Sample name	Extracted mass (g)
B04-2	0.1172
B04-3	0.1473
B04-4	0.1038
C11-1	0.1259
C11-2	0.1239
C11-3	0.0981
C03-1	0.1246
C03-2	0.1285
C03-3	0.1206
C05-1	0.0273
C05-2	0.0574
C05-3	0.0633
C06-1	0.0212
C06-2	0.0165
C06-3	0.0178
B09-1	0.1153
B09-2	0.059
B09-3	0.0854
B12-1	0.1569
B12-2	0.1205
B12-3	0.2471
P01-1	0.1647
P01-2	0.1465
P01-3	0.1193
P02-1	0.0981
P02-2	0.0979
P02-3	0.0659
P03-1	0.1269
P03-2	0.1118
P03-3	0.0584

Supplementary Table S6. DNA concentrations following extraction. “Too High” means DNA concentrations exceeded the range of the Qubit 4 fluorometer.

Sample type	Sample name	DNA concentrations (ng/ul)		
		A	B	C
Aerosol	Arena	3.4	-	-
	Small Lake	0.96	-	-
	PCSP	9.58	-	-
	Allen Bay	4.04	-	-
	North Lake	6.94	-	-
	Teardrop	8.36	-	-
	Char Lake	3.06	-	-
Lake water	Char Lake 2m	4.98	11.00	6.17
	Char Lake 7m	15.30	16.70	13.20
	Char Lake 15m	3.38	15.10	4.24
	Meretta Lake 2m	2.32	9.01	18.50
	Meretta Lake 6m	11.00	12.90	3.58
	Resolute Lake 2m	16.90	12.70	18.10
	Resolute Lake 10m	16.30	15.70	26.30
	Resolute Lake 15m	18.70	4.45	13.70
Microbial Mats	B04	Too High	10.3	Too High
	C11	41.3	14.7	11.2
	C03	Too High	Too High	Too High
	C05	6.74	9.75	Too High
	C06	54.0	34.9	39.4
	B09	10.4	21.0	9.06
	B12	Too High	16.7	Too High
	P01	43.6	54.0	38.9
	P02	60.0	19.5	Too High
	P03	17.2	15.8	11.8

Supplementary Table S7. Presence (red) and absence (white) of specific antibiotic resistance genes encoding resistance to families reported in Table 1 across samples. degree of impact and habitats.

	High impact						Mid impact						Low impact								
**Antibiotic family | ARG**	**Meretta Lake**	**B09 Mat**	**B12 Mat**	**C11 Mat**	**C03 Mat**	**B04 Mat**	**Resolute Lake**	**C05 Mat**	**C06 Mat**	**Tear drop aero**	**Arena aero**	**Resolute Bay aero**	**Char Lake**	**P01 mat**	**P02 mat**	**P03 mat**	**Allen Bay aero**	**North Lake aero**	**PCSP aero**	**Small Lake aero**	**Char Lake aero**
Aminoglycoside | A16S	■	■	■	■	■	■	■	■	■		■		■	■	■	■					
Aminoglycoside | rpsL			■	■	■	■	■						■	■		■					
Aminoglycoside | rrsA	■		■	■	■	■	■						■								
Aminoglycoside | rrsC			■	■		■															
Aminoglycoside | rrsH			■	■		■									■						
Aminoglycoside | AAC(3)				■																	
Aminoglycoside | AAC(6')				■		■															
Cationic antimicrobial peptide | (CAP16S)				■		■									■						
Fluoroquinolone | gyrA	■		■	■		■							■								
Macrolide | MLS23S	■	■	■	■	■	■	■						■	■	■	■					
Aminocoumarin | parE				■																	
Rifampin | rbpA			■	■			■														
Rifampin | rpoB			■	■	■	■										■					
Mycobacterium tuberculosis | rpsA			■	■																	
Mycobacterium tuberculosis | thyA				■																	
Tetracycline | TET16S	■		■				■				■		■								
Elfamycin | tufAB	■	■	■	■	■	■	■	■						■	■	■					

Supplementary Figure

Supplementary Figure S1. Principal coordinate analysis of microbial communities by habitat type overlayed with ARG hit vectors using log-transformed Bray-Curtis dissimilarity matrix. MLS: macrolides, lincosamindes, streptogramines; Tetra: tetracycline; Fluoro: fluoroquinolone; Catio: cationic peptides; Tuber: *M. tuberculosis* drugs; Elfa: elfamycins; Amigly: aminoglycosides.

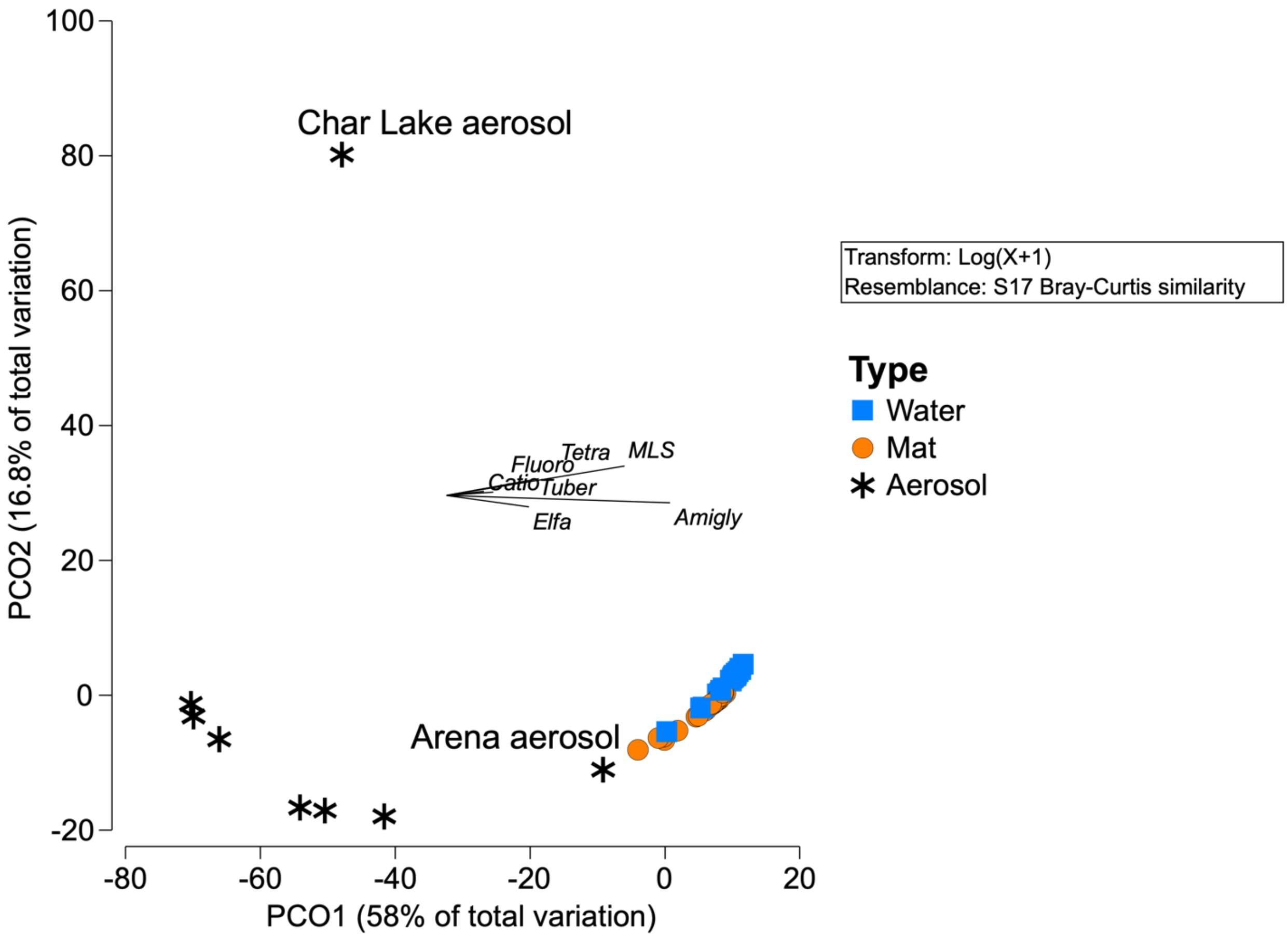

Supplement: Supplementary file 1 [file qvae021_Supplemental_File.pdf]
